# Microglia Acquire Distinct Activation Profiles Depending on the Degree of α-Synuclein Neuropathology in a rAAV Based Model of Parkinson's Disease

**DOI:** 10.1371/journal.pone.0008784

**Published:** 2010-01-20

**Authors:** Vanesa Sanchez-Guajardo, Fabia Febbraro, Deniz Kirik, Marina Romero-Ramos

**Affiliations:** 1 Central Nervous System Disease Modeling Group, Department of Medical Biochemistry, Aarhus University, Aarhus, Denmark; 2 Department of Experimental Medical Science, Brain Repair and Imaging in Neural Systems, Lund University, Lund, Sweden; National Institutes of Health, United States of America

## Abstract

Post-mortem analysis of brains from Parkinson's disease (PD) patients strongly supports microglia activation and adaptive immunity as factors contributing to disease progression. Such responses may be triggered by α-synuclein (α-syn), which is known to be the main constituent of the aggregated proteins found in Lewy bodies in the brains of PD patients. To investigate this we used a recombinant viral vector to express human α-syn in rat midbrain at levels that induced neuronal pathology either in the absence or the presence of dopaminergic cell death, thereby mimicking early or late stages of the disease. Microglia activation was assessed by stereological quantification of Mac1+ cells, as well as the expression patterns of CD68 and MCH II. In our study, when α-syn induced neuronal pathology but not cell death, a fast transient increase in microglia cell numbers resulted in the long-term induction of MHC II+ microglia, denoting antigen-presenting ability. On the other hand, when α-syn induced both neuronal pathology and cell death, there was a delayed increase in microglia cell numbers, which correlated with long-lasting CD68 expression and a morphology reminiscent of peripheral macrophages. In addition T-lymphocyte infiltration, as judged by the presence of CD4+ and CD8+ cells, showed distinct kinetics depending on the degree of neurodegeneration, and was significantly higher when cell death occurred. We have thus for the first time shown that the microglial response differs depending on whether α-syn expression results on cell death or not, suggesting that microglia may play different roles during disease progression. Furthermore, our data suggest that the microglial response is modulated by early events related to α-syn expression in substantia nigra and persists at the long term.

## Introduction

In the majority of cases, the etiology of sporadic Parkinson's disease (PD) still remains largely unknown. However, emerging evidence suggests that multiple factors, both genetic and acquired, contribute to neurodegeneration of the dopaminergic cells of the substantia nigra (SN) in these patients [for review see [1], [2]]. There are several lines of study indicating that activation of microglia, with subsequent production of pro-inflammatory cytokines, aggravates the neurodegenerative process in PD [3], [4]. *In vivo* imaging studies using PET ligands have shown microglial activation in patients with PD [5], [6]. Here, not only the number of activated microglia cells is increased, but molecules related to inflammation are elevated as well. In the nigro-striatal system, pro-inflammatory cytokines, such as tumor necrosis factor (TNF)-α, interleukin (IL)-1β and interferon (IFN)-γ, are increased. These have been found to co-localize with microglia in histological data, and are increased in serum of PD patients. [7], [8], [9], [10], [11], [12]. Importantly, these cytokines can act directly on dopaminergic cells and lead to activation of caspases [13], [14], [15], [16]. Last but not least, microglia activation can lead to free-radical formation, which can contribute to the increase in oxidative markers found in PD [17]. Taken together, the data suggest that the persistent activation of microglial cells is dynamically involved in the disease's progression. Although it is tempting to assume that this event could contribute to neuronal damage and cell death in PD, it is also possible that the microglia population exerts a protective effect on neurons in the SN, thus delaying neurodegeneration progression.

Another determining factor in PD etiology is α-synuclein (α-syn). Missense mutations in the α-syn gene have been identified to cause autosomal dominant familial PD [18], [19], [20]. Furthermore, the multiplication of the α-syn gene leads to PD, indicating that a mere over expression of the protein can lead to dopaminergic cell death [21], [22], [23], [24]. In both familial and non-familial cases, where no genetic mutations are found, α-syn is the major component of Lewy bodies (LBs) [25]. The relationship between α-syn pathology and activation of microglia remains poorly studied. It is however possible, that α-syn plays a role in microglia activation as shown by several *in vitro* studies [26], [27], [28]. Zhang and colleagues showed that depletion of microglia diminished the dopaminergic cell death induced by exposure to aggregated α-syn in a cell culture system [29]. Nitrated α-syn has been suggested to play an important role in microglia mediated inflammatory response in PD [30], [31], [32] as well as the induction of immunity [33].

Although the presence of neuroinflammation in PD has long been accepted, the contribution of the adaptive immune system is still poorly defined. Several findings in PD patients support its role in the disease process: the presence of T cells in the SN of patients; the existence of IgG that react with dopaminergic tissue; and dopamine derived oxidative products in serum and CSF of PD patients [34], [35], [36]. Class I and II major histocompatibility complex (MHC), essential molecules for antigen presentation, are increased in the striatum and SN, respectively, of PD patients [14], [37]. Indeed, correlation between MHC class II antigen expression and α-syn deposition has been observed in post-mortem nigral specimens from PD patients [38]. Moreover, antibodies against α-syn were found in patients suffering from familial PD [39]. Together suggesting that α-syn may be the antigen that induces the immune response.

In the present study we use a recently described progressive neurodegeneration model of PD based on over-expression of human wild type (wt) α-syn mediated by injection of recombinant adeno-associated virus (rAAV) directly into the SN [40], [41], [42]. We have investigated the temporal profile of microglia activation as a function of α-syn neuropathology and its relationship with nigral dopaminergic cell death. We designed an *in vivo* experimental approach, where animals expressed α-syn in the nigro-striatal pathway at such levels as to achieve pathological mishandling of α-syn that resulted in significant cell death in SN. In parallel, a second group of animals expressed levels of α-syn that resulted in pathological accumulation of α-syn and nigro-striatal degeneration but where cell death in SN was absent. Microgliosis and expression of activation related molecules were analyzed at different time points. Our data suggest that α-syn expression not only leads to persistent microglia activation, but that depending on the degree of induced neuropathology distinct microglial responses will occur. α-Syn expression levels capable of inducing dopaminergic cell death correlate with the long-term induction of macrophagic microglia; whereas, at levels where only neurodegeneration is taking place, we have microglia with antigen presenting capabilities.

## Methods

### Animals

Adult female Sprague Dawley rats (Taconic, Denmark) of 225–250 g (at the time of surgery) were housed three to a cage with *ad libitum* access to food and water during a 12 hr light/dark cycle. All procedures were approved by the Ethical Committee for the use of laboratory animals in Aarhus University.

### Viral vectors

The vectors contain the transgene of interest [human wt α-syn, or the enhanced green fluorescent protein (GFP)] under the control of the synthetic chicken beta actin (CBA) promoter [40], [41], [43]. rAAV2/5 vectors were purified by iodixanol step gradients and ion-exchange as described in detail elsewhere [44]. The final titers of the concentrated vector stocks were as follows: a first rAAV2/5-wild type α-syn (6.2×10^13^ genome copies/ml), a second rAAV2/5-wild type α-syn (6.7×10^13^ genome copies/ml), and rAAV2/5-GFP (7.9×10^12^ genome copies/ml) as determined by quantitative PCR.

### Surgical procedure

Surgery was conducted under anesthesia using a stereotaxic frame (Stoelting, Wood Dale, IL, USA) and a 5 µl Hamilton syringe fitted with a glass capillary (outer diameter of 60–80 µm). Animals received a single 2 µl injection of either first, second rAAV2/5-α-syn or rAAV2/5-GFP into the right SN at the following coordinates: 5.2 mm posterior, 2.0 mm lateral to bregma, and 7.2 mm ventral relative to dura. Vector stocks were injected at a rate of 0.2 µl/30 s. The needle was left in position for an additional 5 mins after the infusion was completed before being slowly retracted. Animals were then sutured with metal clips and returned to their cage where food and water were freely available.

### Immunohistochemistry

Under pentobarbital anesthesia the animals were perfused through the ascending aorta with physiological saline, followed by 4% ice-cold para-formaldehyde. The brains were post-fixed in the same solution for 2 hr, transferred to 25% sucrose, and sectioned on a freezing microtome at 35–40 µm in the coronal plane. Immunohistochemical stainings were performed on free-floating sections using the following antibodies: anti human α-syn (rabbit, against epitope 116–131, 1∶4000); and of mouse origin: tyrosine hydroxylase (TH) (Chemicon, Temecula, CA, 1∶2000), ED1 (CD68, Serotec, 1∶200), Mac1 (CD11b, Serotec, 1∶500), MHC II (Serotec, 1∶250), CD3 (Serotec, 1∶500), CD8α (Serotec 1∶500) and CD4 (domain 1, Serotec, 1∶500). The sections were quenched for 10 min in a solution of 3% hydrogen peroxide/10% methanol. Sections were rinsed three times in potassium-phosphate buffer (KPBS) between each incubation period. All incubation solutions contained 0,25% Triton X-100 in KPBS. One hour of pre-incubation with 5% appropriate normal serum was followed by overnight incubation at room temperature of the primary antibody in 2,5% normal serum. Thereafter the sections were incubated for 2 hr with the appropriate biotinylated secondary antibody (1∶200, Vector Laboratories, Burlingame, CA) in 1% normal serum, followed with 1 hr incubation with avidin-biotin-peroxidase complex in PBS (ABC Elite, Vector Laboratories, Burlingame, CA). Visualization was done using 3,3-diaminobenzidine and 0.01% of hydrogen peroxide for TH and α-syn visualization, and 0.001% for the others. The sections were mounted in chrome-alum-coated glass slides and cover-slipped. During cover-slipping some sections were counter-stained with cresyl blue.

### Stereological quantification of cell numbers in the midbrain

The unbiased stereological estimation of the total number of TH+ cells in SN was made using the optical fractionator as previously described [41]. This sampling technique is not affected by tissue volume changes and does not require reference volume determinations. Sampling was done using the NewCAST software from Visiopharm. A low power objective lens (1.25x, SPlan) was used to delineate the borders of the SN at all levels in the rostrocaudal axis. The medial border of the SN and lateral border of the VTA was defined by a vertical line passing through the medial tip of the cerebral peduncle (and by the medial terminal nucleus of the accessory nucleus of the optic tract, when present in the sections). Ventral border followed the dorsal border of the cerebral peduncle, thereby including the TH-positive cells in pars reticulata (SNr), and the area extended laterally to include the pars lateralis in addition to the pars compacta (SNc). The sections used for counting covered the entire SN from the rostral tip of the pars compacta back to the caudal end of the pars reticulata. This typically yielded 8–9 sections in a series. The counting frame was placed randomly on the first counting area and systematically moved through all counting areas until the entire delineated region was sampled. The sampling frequency was chosen by adjusting the X-Y step length between 100 and 200 µm, so that about 100–200 TH-positive cells were counted in each side of the SN. Actual counting was done using a 40x objective (NA 0.75). The estimates of the total number of neurons were calculated according to the optical fractionator formula and a coefficient of error <0.10 was accepted [45], [46].

For the microglia quantification, similarly adjacent (7–8) serial sections were used. An observer blind to the samples identity quantified the number of Mac1 positive cells in the SN as described above (Leica microscope at a 40x magnification). Here the X-Y step length used was between 300–400 µm in order to count 100–200 Mac1+ cells in each side of the SN. A positive cell was defined as a nucleus covered and surrounded by Mac1 immunostaining.

### Morphological characterization

During the stereological analysis, the morphology of each Mac1+ cell was scored according to the length and thickness of their processes, the characteristics of their cell body and the look of the nucleus. Four cellular profiles were defined: Type A, cells with no visible cytoplasm, a round dense nucleus, and with long thin processes with little branching; these were deemed “resting” microglia. Type B, cells with a visible thin cytoplasm surrounding a dense nucleus; processes are very long and thin, with many branches of less defined edges. Type C, cells with elongated and irregular body, enlarged and less defined nucleus, and with shorter re-defined processes of varying thickness and little branching. These cells are reminiscent of antigen presenting cells. Type D, have a big cell body merging with the processes, the nucleus occupies most of the cell body and is not always distinguishable; processes are few thick and short. These cells look indistinguishable from peripheral macrophages. (See results section for further description)

### Microscope analysis of brain sections

An observer blind to the origin of the section, analyzed one section from the striatum and one from SN of each animal per antibody tested. For MHC II and ED1 the analysis was done at 10x and 40x and the extension of the marking was scored as described in the results section. For CD3, CD4 and CD8, the observer counted the number of positive cells within the section at 20x and 40x.

### Statistics

Statistical comparison of data was performed using JMP statistical software v. 5.01 (SAS Institute Inc. Cary, NC, USA). Time and group interactions were analyzed using two-way factorial ANOVA, when significant followed by Tukey HSD post-hoc analysis. When appropriate, individual time points within groups were compared by one-way ANOVA followed by Tukey-Kramer or Dunnet's post-hoc analysis. For statistic analysis on scores (MHCII and CD68) non-parametric oneway ANOVA followed by Van der Waerden post-hoc analysis was performed.

## Results

### α-Synuclein induces neurodegeneration and dopaminergic cell death

In order to study the role of microglia as a function of human α-syn expression, we decided to follow the microglial response in two different scenarios: one where α-syn expression did not lead to dopaminergic cell loss, but only to pathological accumulation of the protein and striatal fiber loss; another where α-syn induced a progressive cell death in the SN.

**Figure 1:**
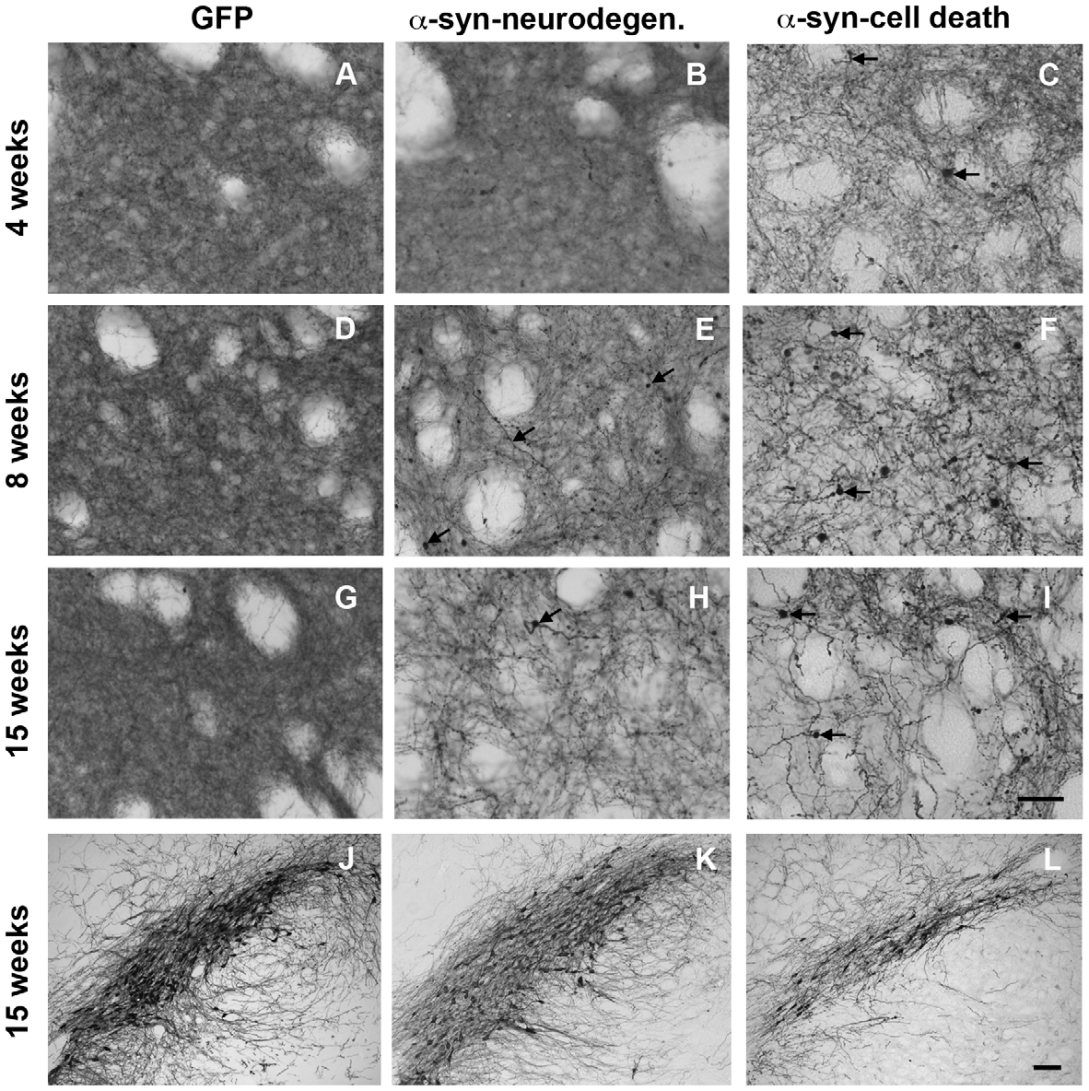
Tyrosine hydroxylase immunostaining. TH immunohistochemical staining of striatal (**A-I**) and nigral sections (**J-L**) from animals of the GFP group (**A, D, G** and **J**), the α-syn-neurodegeneration group (**B, E, H** and **K**), or the α-syn-cell death group (**C, F, I** and **L**) at 4 weeks (**A-C**), 8 weeks (**D-F**) or 15 weeks (**G-L**) post- rAAV2/5 injection. GFP expressing animals showed dense dopaminergic fiber staining in striatum (**A, D** and **G**) and numerous TH+ neurons in SN (**J**) that did not change after 15 weeks. Animals in the α-syn-neurodegeneration group showed an apparent decrease in TH+ fiber staining in striatum at 8 weeks that further decreased at 15 weeks (**E** and **H**). After 8 weeks, numerous TH+ fibers appeared thicker and/or with small round formations, which became bigger after 15 weeks (arrows in **E** and **H**). However, the number of TH+ neurons in SN (**K**) remained similar to control levels (both contralateral side (not shown) and to GFP in **J**) after 15 weeks. In the cell death group α-syn expression lead to loss of TH fibers that was already apparent at 4 weeks; with time fibers became thicker and more apparent, although less numerous (**C, F** and **I**). TH+ fibers in these animals showed thickening and pathological round formations from early time points that increased with time (arrows in **C, F** and **I**). This was accompanied by a significant decrease of TH+ neurons of similar magnitude at 4, 8 (data not shown) and 15 weeks (**L**). Scale in I: 40 µm, applies to **A**-**I**. Scale in L**:** 100 µm, applies to **J-L.**

**Figure 2:**
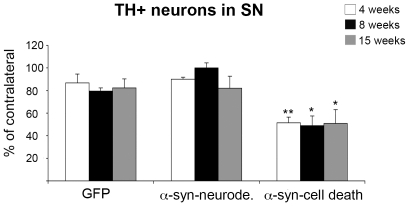
Dopaminergic cell survival in Substantia Nigra. Graph illustrates the estimation of total dopaminergic neurons in SN (expressed as % of control, contralateral side values) using stereological tools in all groups and time points. A significant decrease of the number of TH+ cells was observed in the α-syn-cell death group at all time points. Two-way ANOVA [F (8,46) = 6.18, p<0.01] followed by individual contrast. One-way ANOVA: * <0.05 and ** <0.01 different from the GFP group at the same time point, effect of group at 4 weeks [F (2, 14) = 9.3, p<0.01], at 15 weeks [F (2, 16) = 9.3, p<0.01], at 15 weeks [F (2, 14) = 4.1, p<0.05] followed by a Dunnett's post-hoc analysis.

To confirm our experimental design we performed stereological quantification of TH+ neurons in SN in all groups at 3–4, 8 and 15 weeks. As anticipated, one group of animals, termed α-syn-neurodegeneration, did not show any significant loss of dopaminergic cells when compared to GFP control animals (Figure 1J & K and Figure 2). However, the other group, termed α-syn-cell death, showed significant cell death as soon as 4 weeks that remained unchanged at 8 and 15 weeks (Figure 1L and Figure 2). At striatal level, dopaminergic fiber density decreased as expected in these animals. The remaining fibers appeared thicker and with big TH+ round formations that increased progressively (Figure 1C, F & I). Interestingly, in the animals where α-syn failed to induce neuronal cell loss, we observed a decrease in fiber density and progressive pathological formations (Figure 1B, E & H). In the control GFP animals, no change was observed as compared to the contralateral side (Figure 1A, D & G).

The criterion followed here to match the two α-syn vectors was the rAAV titer, i.e. the numbers of viral particles per ml of each rAAV batch. However, the level of transgenic protein expression per infected cell will depend not only in the number of physical particles injected, but also in the ability of these particles to infect the cell and express the protein (viral infectivity). This means that despite having similar titers, two vector batches produced in two different production runs may result in different infectivity per physical particle, potentially leading to dissimilar expression levels. Therefore, it is possible that two vectors with similar physical titer result in one case in an α-syn expression level that induced cell death while in the other case merely protein accumulation but not frank cell loss.

### α-Synuclein induces pathological formations in the striato-nigral neurons independently of neuronal death

**Figure 3:**
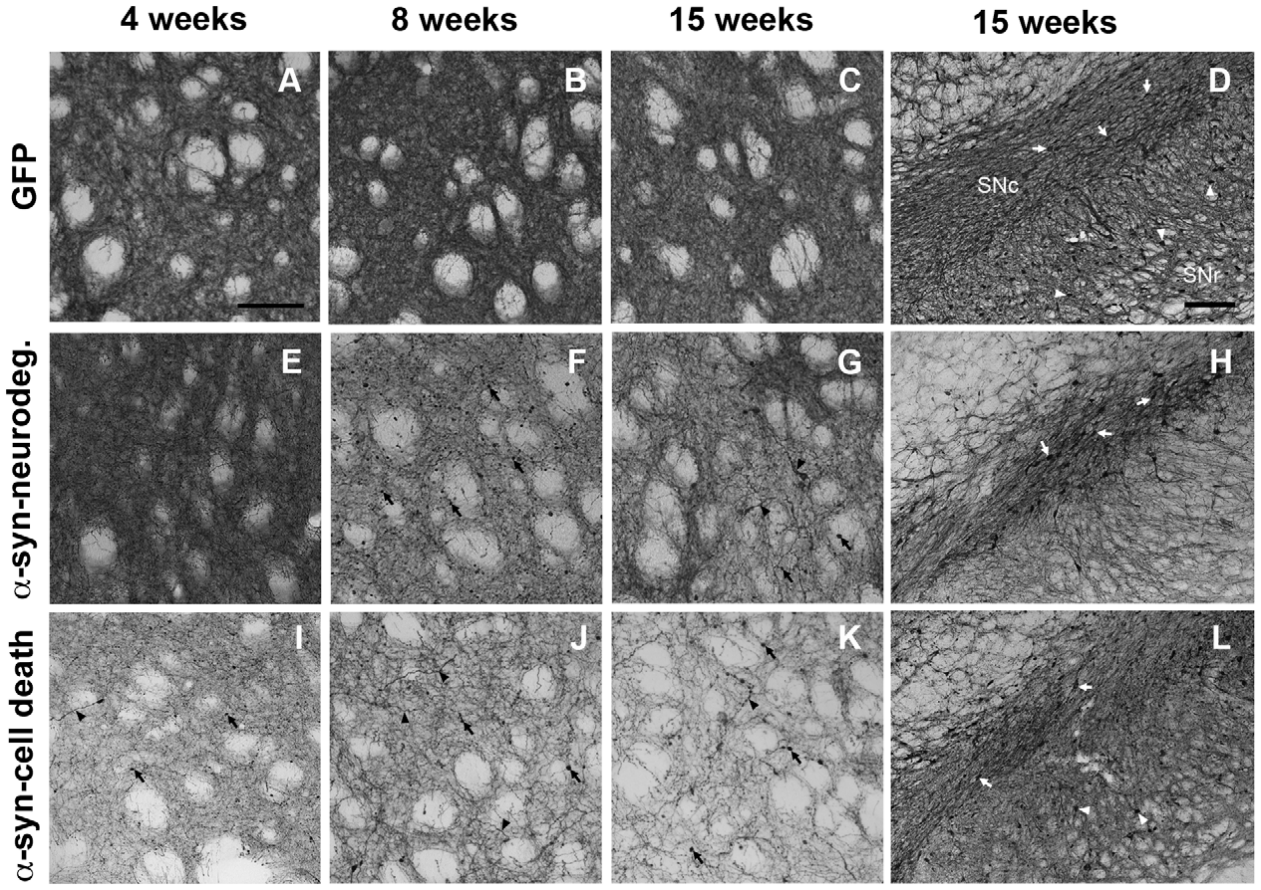
Transgene expression. Transgene immunostaining at striatal (**A**–**C**, **E**–**G** and **I**–**K**) or nigral level (**D**, **H** and **L**) from animals of the GFP group (**A**–**D**), α-syn-neurodegenerative group (**E**–**H**), and α-syn-cell death group (**I**–**L**) at 4 weeks (**A, E** and **I**), 8 weeks (**B, F** and **J**), or 15 weeks (**C, D, G, H, K** and **L**) post- rAAV2/5 injection. GFP immunostaining was observed in the ipsilateral side of animals injected with GFP-rAAV2/5, as a dense fiber staining where no apparent pathology was observed (**A, B** and **C**). In the midbrain we observed neurons expressing GFP both in SNc (arrows) and SNr (arrowheads) that remained after 15 weeks (**D**). Animals in the α-syn-neurodegeneration group showed dense fiber immunostaining for human-α-syn at 4 weeks (**B**). Fiber staining decreased progressively with the concomitant appearance of pathological formations (**F** and **J**). At 8 weeks numerous round α-syn+ formations were observed (arrows in **F**). With time these formations became bigger (arrows in **J**) and thickening of α-syn+ fibers was observed (arrowheads in **J**). At the nigral level, numerous α-syn+ neurons in SNc persisted after 15 weeks (arrows in **H**). In animals of α-syn cell death group, fiber staining was lower than in the other α-syn group at 4 weeks (**I** vs. **E**). Progressively α-syn+ fibers became thicker and less abundant in striatum (arrowheads in **I**–**K**). In addition, α-syn+ pathological formations were already apparent at 4 weeks, they persisted and became bigger as time progressed (arrows in **I**–**K**). In the midbrain we observed α-syn+ neurons both in SNc (arrows) and SNr (arrowheads) that remained after 15 weeks (**L**). There was a lower number of α-syn+ neurons in SNc than in the α-syn-neurodegeneration group (**L** vs. **H**). Scale: 100 µm, in A applies to A–C, E–G and I–K; in D applies to D, H and L.

Local injection into the midbrain of rAAV-α-syn resulted in transduction of nigral dopaminergic cells and expression of human α-syn throughout the nigro-striatal system as expected (Figure 3E-L). Regarding the nigral dopaminergic neurons, animals in the α-syn neurodegeneration group displayed after 4 weeks numerous α-syn+ cell bodies that were confined to the SNc, these persisted at 15 weeks (Figure 3B & H). In addition dense fiber network immunostained for human α-syn was observed in striatum (Figure 3E). Starting at 8 weeks, these fibers showed small pathological accumulations that increased in occurrence and thickness with time, while fiber density progressively decreased (Figure 3E-G). In comparison, animals with apparent dopaminergic cell death exhibited fewer α-syn+ cells in the SNc at all time points, presumably due to the significant cell loss observed at 4–15 weeks (Figure 3L). In this group, the density of α-syn+ fibers was lower at all time points and as soon as 4 weeks, numerous pathological α-syn+ accumulations and thickening of the striatal terminals were observed (Figure 3I). Although the number of α-syn+ cells in SN remained unchanged at all points, fibers in striatum decreased with time, with the concomitant pathological accumulation of α-syn in the remaining fibers (Figure 3J & K). GFP expressing animals showed a strong GFP immunostaining in cells of the SNc that persisted at 15 weeks (Figure 3D); as well as, dense fiber immunostaining at striatal level that did not decrease with time nor showed any pathological abnormal accumulation (Figure 3A–C).

We have thus generated two sets of animals where we observed progressive nigrostriatal degeneration and α-syn accumulation, but where robust cell death was only induced in one of them. We will refer hence to these groups as α-syn-neurodegeneration and α-syn-cell death.

### Modulation of microglia morphology and cell number in Substantia Nigra is dependent on the degree of α-synuclein induced pathology

**Figure 4:**
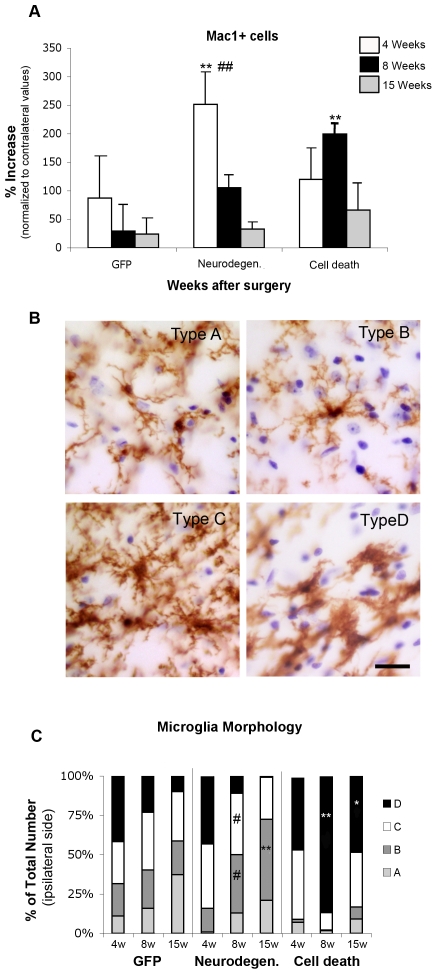
Microglia morphology and cell number. (**A**) Animals expressing GFP, or α-syn at levels that caused either neurodegeneration or cell death were killed at 4 (white bars), 8 (black bars) and 15 (grey bars) weeks after surgery. SN resident microglia cell numbers and morphology were stereologically analyzed at each time point. Data represents average increase of Mac1+ microglia cell number in the ipsilateral SN as compared to the contralateral SN (n = 5–6) + S.E.M. Two-way ANOVA [F (8,45) = 11.2, p<0.0001] followed by Tukey HSD post-hoc analysis. * or # p<0.05 ** p<0.01 (*) compared to the GFP control group at same time point; (#) compared to the other α-syn group at same time point. (**B**) The four morphologies of microglia are represented: Type A, corresponding to resting microglia: cells with a round dense nucleus without a visible cytoplasm surrounding it, and with long thin processes. Type B: there is a visible small thin cytoplasm around a dense nucleus, processes remain thin, but are longer with many branches of less defined edges. Type C: the cell body becomes elongated and irregular, with an enlarged and less defined nucleus, processes become shorter of varying thickness and little branching. Type D: the cell body is big and dark, merging with thick short processes; the nucleus occupies most of the cell body and is not always distinguishable. Scale in type D: 20 µm, applies to all. (**C**) Stereological quantification of each morphology type is depicted as the average percentage distribution per group as a function of time. Two-way ANOVA, when significant followed by Tukey-Kramer post-hoc analysis. # p<0.05 compared to the other α-syn group at same time point; * p<0.05, ** p<0.01 compared to the other two groups at same time point.

The microglia population in midbrain was analyzed by stereological quantification of Mac1+ cells at all time-points. Numbers were normalized to the number of microglia in the contralateral uninjected side of each animal. Only cells where a nucleus (counterstained with cresyl violet) was completely covered and surrounded by Mac1+ staining were considered positive cells. Four weeks after surgery there was an increase in microglia cell numbers in the injected SN in all groups. However, the increase was significantly higher in the α-syn-neurodegeneration group (Figure 4A), where numbers gradually decreased to control levels at 8–15 weeks. In contrast, when cell death occurred, microglia cell numbers peaked at 8 weeks, to eventually drop significantly and return to basal levels at 15 weeks (Figure 4A). It is interesting to note that all the animals of α-syn-cell death group had weak Mac1+ staining at 4 weeks.

During the stereological analysis we further quantified microglia morphologically. Four cellular profiles (Figure 4B) were defined according to the length and thickness of their processes, the characteristics of their cell body and the look of the nucleus as detailed in Material and Methods. Type A, were deemed “resting” microglia as they were the dominant type of microglia in the contralateral side of the brain, as well as brain areas other than the SN. Type B, were usually found isolated, the end of their processes in association with many Mac1- nuclei. Type C, were often seen in association with blood vessels, some of them were clearly inside the lumen of the vessel (Figure S1C & D). They usually had Mac1- nuclei in direct contact with their processes but seldom with the cell body; and although often close in space to each other, they never formed clusters. These cells are reminiscent of antigen presenting cells. Type D, were seldom found isolated or in contact with blood vessels, they tended to form clusters and always had multiple nuclei associated with the cell body (visible at different focus planes, data not-shown). These cells look indistinguishable from peripheral macrophages.

We observed that in all groups at 4 weeks type D (macrophagic) cells constitute over 40% of the microglia population (Figure 4C). At 8 weeks the most distinctive event is the significant prevalence of type D microglia in the α-syn-cell death group, whereas both GFP and α-syn-neurodegeneration show similar profile distribution (Figure 4C). At long term, in the α-syn-cell death group, type D (48%) still constitutes the most abundant microglia, while in the α-syn-neurodegeneration animals, type B constitutes over 50% of the population. At this time point, 37% of the microglia in GFP have returned to their resting stage (Type A, Figure 4C).

### Microglia up-regulates distinctive activation markers depending on the degree of α-synuclein induced neurodegeneration

**Figure 5:**
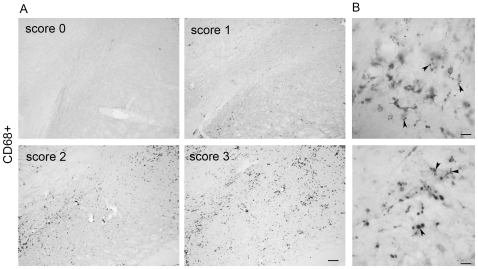
CD68 microglial expression. The levels of CD68 expression were scored 0–3 (**A**): Score 0, non or 1–3 isolated positive cells; Score 1, few scattered cells; Score 2, throughout the SNc; Score 3, throughout the SN. Scale in Score 3: 80 µm, applies to all in A. (**B**) Higher magnification of representative CD68+ immunostaining. Notice how the intracellular vesicular localization of the protein (arrowheads) makes it difficult to determine how many positive dots belong to one microglia. Scale in B: 13 µm (top) and 20 µm (bottom).

To correlate morphology to function, we studied the levels of expression of CD68 (ED1) as a marker for macrophagic function, and MHC II for antigen presentation ability (Table 1), both being practically undetectable in naïve/healthy animals. We assessed the level of up-regulation of CD68, where 3 scores were given based on the number of CD68+ cells and their localization (Figure 5). In the GFP group, at 4 weeks we observed CD68 positive staining of varying degree (Figure 5 & Table 1), but CD68 expression was absent at 8–15 weeks, suggesting that the unspecific response to the viral vector injection was resolved. In the α-syn–neurodegeneration group, at 4 weeks, CD68+ microglia were mainly found scattered throughout the SNc. At this point GFP animals showed significantly more CD68 expression, suggesting that viral vector injection and infectivity plays a role in the initial response (Table 1). At later time points, differing from the GFP group, some animals in α-syn-neurodegeneration still showed scattered CD68+ cells, though this was not significant (Table 1). In contrast, when α-syn expression led to cell death, there was a significant up-regulation of the macrophagic marker at 4 weeks, where we observed an intense dense punctuated staining throughout the whole SN. Expression persisted with time, but became restricted to the SNc at 8 weeks (Table 1 & Figure 5A). Due to the intracellular vesicular localization of the CD68 receptor, it was not possible to directly assess the morphology of the cell expressing it by means of its staining pattern (Figure 5B). However, its high up-regulation correlates with SN where the predominant microglial morphology is of type D.

**Table 1:** Analysis of expression levels of microglia activation markers induced by α-synuclein neuropathology in Substantia Nigra.

	4 weeks	8 weeks	15 weeks
**CD68**			
***GFP***	2.00±1.58 ^#^	0.00±0.0	0.00±0.0
***Neurodegeneration***	0.00±0.45	0.00±0.45	0.00±0.45
***Cell death***	2.00±0.53 ^#^	2.50±1.28 ^##, **^	2.00±0.53 ^##, **^
**MHC II**			
***GFP***	2.00±1.58	1.00±1.58	0.00±1.05
***Neurodegeneration***	4.50±0.91^*^	4.00±0.91^*^	2.50±0.91
***Cell death***	4.00±0.53	4.00±1.28^*^	3.00±2.10

An observer blind to the sections identity scored expression levels based on number of positive cells and area covered, as explained in Figure 3. CD68: 0–3, MHC II: 0–5. Numbers are median ±95% confidence interval. Statistical analysis: non-parametric oneway ANOVA followed by Van der Waerden post-hoc analysis. * or # p<0.05, ** or ## p<0.01 (*) compared to GFP control, (#) compared to the neurodegeneration group.

**Figure 6:**
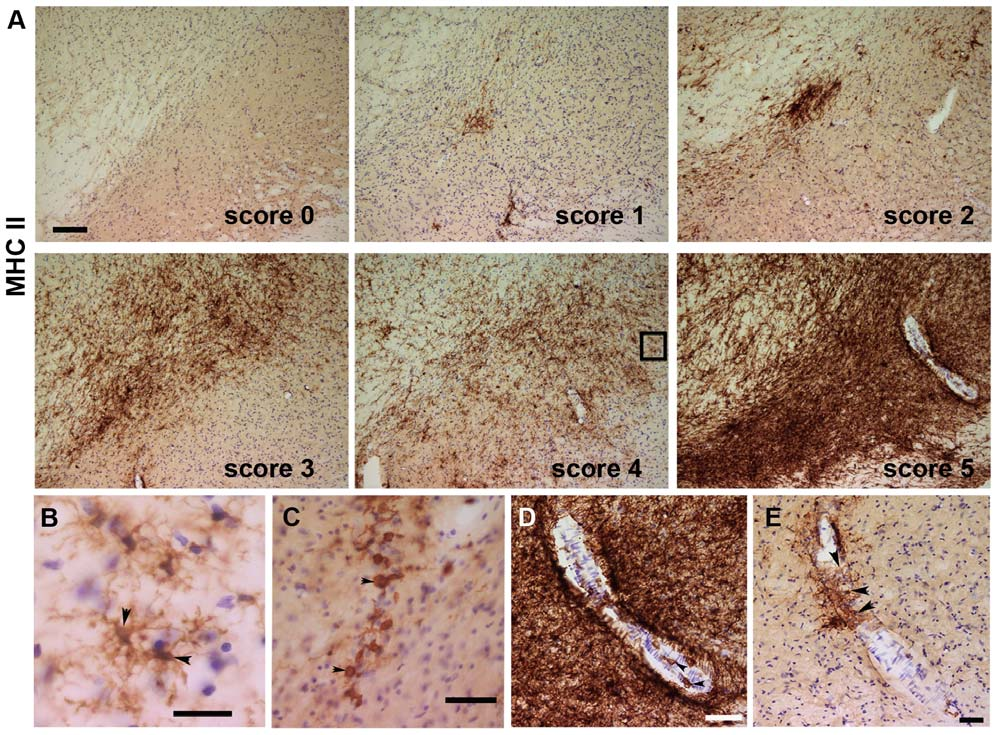
MHC II microglial expression. Photos show representative nigral sections immunostained for MHCII. The level of MHC II expression in microglia was scored 0–5 (**A**): Score 0, no positive cells; Score 1, few isolated cells; Score 2, scattered throughout the SNc or few clusters; Score 3, throughout the SNc; Score 4, throughout the SN; Score 5, saturation of the SN. (**B**) High magnification photo of the insert showed in A Score 4. The most abundant MHC II+ microglia morphology corresponded to microglia type B (arrow). (**C**) In the groups expressing α-syn, we also observed numerous small MHC II+ round cells at 4 (not shown) and 8 weeks. (**D**–**E**) MHC II+ cells in association with blood vessels. Occasionally we saw MHC II+ cells, which seemed to be inside of the vessel's lumen (arrowhead in **D**); in other cases MHC II+ cells seemed to penetrate and/or surround the vessel's wall (arrowheads in **E**). The images come from, C and D: neurodegeneration at 4 weeks (Score 5), E: neurodegeneration 8 weeks (Score 3). Scale in Score 0: 90 µm, applies to all in A. Scales in B: 10 µm, in C: 40 µm and in D&E: 20 µm.

When MHC II expression was analyzed, all animals in the GFP group showed a varying degree of MHC II+ cells at 4 weeks. Expression was transient, so by 8 weeks, only 2 out of 5 animals showed few MHC II+ cells in SNc (Table 1 & Figure 6A). In animals of the α-syn-neurodegeneration group, we observed at 4 weeks a significant robust up-regulation of MHC II expression in the ventral midbrain, where the area immunostained for MHC II covered the full SN (both SNc and SNr) (Table 1 & Figure 6A). The MHC II+ cells remained numerous throughout the SN at 8 weeks, then decreased and became restricted to SNc at 15 weeks (Table 1 & Figure 6A). In the α-syn-cell death group, MHC II up-regulation was significantly elevated in all animals at 4–8 weeks, although it never reached the levels of expression of the α-syn-neurodegeneration group. Expression remained elevated at the level of the SNc in 3 out of 5 animals at 15 weeks (Table 1 & Figure 6A). MHC II+ microglia exhibited in all groups and at all time points type B morphology (Figure 4B), although at 4 weeks a fraction of the cells exhibited Type C morphology (not shown).

It is interesting to note that we observed round small cells, which stained positive for MHC II (Figure 6C). Although some cells where observed in a fraction of the α-syn-neurodegeneration animals at 4–8 weeks and occasionally in the GFP, these cells were mainly present in the α-syn-cell death group (all animals, all time points).

### Microglia cells associate with blood vessels in α-synuclein expressing animals

During the stereological quantification of serial sections of SN we also noted the appearance of enlarged blood vessels (as compared to those observed in the control side) and whether such vessels where associated to any specific type of cells. Animals of the α-syn-cell death group showed consistently more enlarged blood vessels than the other two groups at 4 weeks (average of 16 enlarged blood vessels vs. 5 per SN). Mac1 low/neg nuclei were associated with and within the blood vessels at this time point (Figure S1A & B); whereas, in the α-syn-neurodegeneration group all associated nuclei were Mac1+ (Figure S1C & D). We always observed Mac1+ Type C cells associated with blood vessels at 8 weeks in both α-syn groups (average of 4 in both groups). At 15 weeks, both α-syn groups had few increased blood vessels with mixed nuclei. In the GFP group we saw few cells, of mixed type, associated with blood vessels at 4 wk (an average of 2 enlarged blood vessel with associated nuclei per SN) and none at 8–15 weeks.

In addition, at 4–8 weeks, we observed MHC II+ cells associated with and within blood vessels in both α-syn groups (Figure 6D & E). In the GFP group this was seen only occasionally and with not specific time correlation (not shown).

### Recruitment of the adaptive immune system depends on the extent of microglia activation

**Figure 7:**
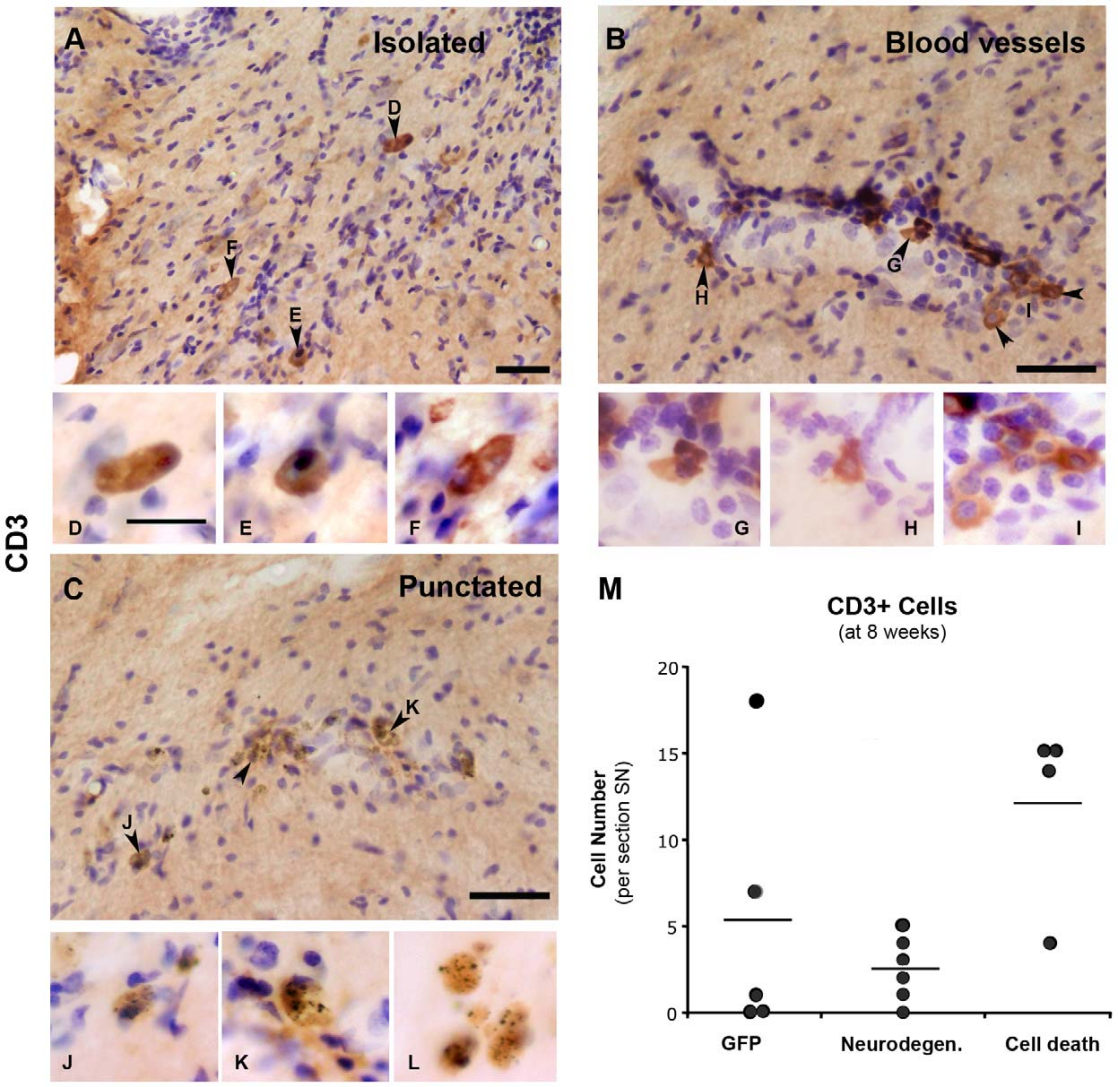
T cell infiltration. Photos show SN of animals immunostained with antibody against CD3 in order to address T cell infiltration (**A**–**L**). CD3+ cells were found either isolated (**A**), associated with blood vessels (**B**) or clustered together (**C**). Immunostaining appeared homogeneously distributed in the cell body (arrowheads in A and B) or punctuated (arrowheads in C). Scale: 40 µm, applies to A–C. The small panels show individual CD3+ cells from A (**D**–**F**), B (**G**–**I**) and C (**J**–**K**) at high magnification. **(L)** Panel shows high magnification of CD3+ cells with punctuated staining without counterstaining in order to appreciate better the staining pattern. Scale: 10 µm, applies to D–L. Photos were taken from sections of animals of the following groups, A: cell death 4 weeks, B: cell death 8 weeks, C+L: neurodegeneration 15 weeks. (**D**) Graph shows average (bar) and individual numbers of CD3+ cells found in one SN section at 8 weeks. These are plotted per animal in each group.

As a specific subset of microglia up-regulate MHC II, pointing to a possible implication of the adaptive immune system, we looked for T cell (CD3+) infiltration into the SN. CD3 is a multimeric membrane kinase that is associated with the T cell receptor (TCR). In our study we observed two patterns of CD3 staining: first, when found isolated or in association with a blood vessel the staining was uniform, giving a round and well-defined staining (Figure 7A–B & D-I). In contrast, when found in clusters, the staining tended to be punctuated, suggesting CD3 clustering, and the individual cell was difficult to determine (Figure 7C & J–L).

Analysis of the number of CD3+ cells per section of SN revealed that T cell infiltration is very variable. In the GFP animals, at all time points, only 2 (out of 5) animals showed CD3+ cells in SN (average ± SEM of numbers in the ipsilateral SN: 4 weeks 7.2±4.4; 8 weeks 5.2±3.4; 15 weeks 5.0±4.2). While in the α-syn-neurodegeneration group, CD3+ cells were observed in half of the animals at 4 weeks, increasing to 5 out of 6 at 8–15 weeks (average ± SEM of numbers in the ipsilateral SN: 4 weeks 2.3±1.9; 8 weeks 2.5±0.7; 15 weeks 5.6±3.4). Interestingly, the presence of CD3+ cells was consistently seen in all animals in the α-syn-cell death group at 4–8 weeks and only one animal of the group did not show T cells at 15 weeks (average ± SEM of numbers in the ipsilateral SN: 4 weeks 6.6±2.6; 8 weeks 12.0±2.6; 15 weeks 5.6±3.5). Indeed at 8 weeks, the number of CD3+ cells was consistently high in this group (Figure 7M, p = 0.0505) and only in this group and at this time point we saw CD3+ cell association with blood vessels. One should note that, in the α-syn-cell death group, staining was mainly of the isolated type, whereas in the α-syn-neurodegeneration group it was of mixed type and in the GFP mainly punctuated.

**Figure 8:**
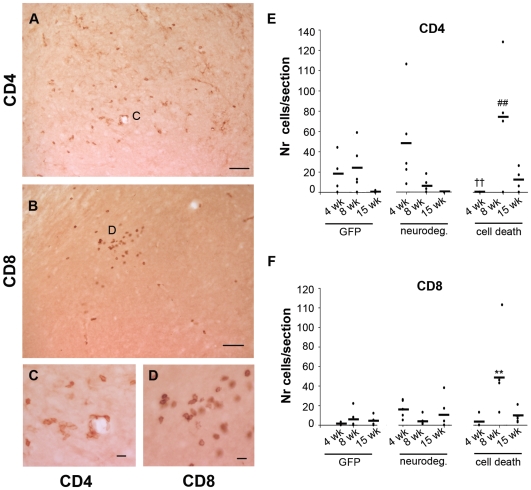
CD4 and CD8 T cell infiltration. Photos show SN sections of an animal of the cell death group immunostained with antibody against CD4 (**A** and **C**) and CD8 (**B** and **D**). The small panels show insets in A (**C**) and in B (**D**) at higher magnification. Scale: 50 µm, applies to A–B, 10 µm applies to C–D. (**E**) Graph shows average (dash) and individual numbers of CD4+ cells found in one SN section per animal of each group plotted per time. Two-way ANOVA [F (8,42) = 4.1, p = 0.001 effect of group and time interaction] followed by Tukey HSD post-hoc analysis. ## or ç p<0.01 (##) compared to the other α-syn group at same time point; (††) different to the next time point of the same group. (**F**) Graph shows average (dash) and individual numbers of CD8+ cells found in one SN section per animal of each group plotted per time. Two-way ANOVA [F (8,41) = 4.3, p = 0.001 effect of group and time interaction] followed by Tukey HSD post-hoc analysis. (**) p<0.01 compared to all other groups at all time point.

T cell infiltration was further analyzed to determine if the observed T cells were helper T cells (CD4+) or cytotoxic T cells (CD8+) (Figure 8). Our analysis revealed that the helper T cell response was of the same magnitude in both α-syn groups, however they were recruited with different kinetics. While α-syn expression in the absence of cell death correlated with an early prominent recruitment of CD4+ cells that disappeared after 15 weeks; in the cell death group, helper T cells peaked at 8 weeks and remained present, although at lower levels, after 15 weeks (Figure 8E). GFP animals showed a moderate infiltration that fully disappeared with time (Figure 8E).

Regarding cytotoxic T cells, we observed a low level infiltration of CD8+ cells in the α-syn-neurodegeneration group at 4 weeks that remained through time (Figure 8F). This response was markedly different in the animals were α-syn induced cell death. Here cytotoxic T cell infiltration was first observed at 8 weeks and it was of significant higher magnitude than the other groups. However, at 15 weeks the numbers of CD8+ T cells were similar in both α-syn groups (Figure 8F). GFP animals showed consistently less CD8+ cells than the α-syn groups at all time points (Figure 8F).

### MHCII+ is up-regulated in the striatum of animals expressing α-synuclein

**Figure 9:**
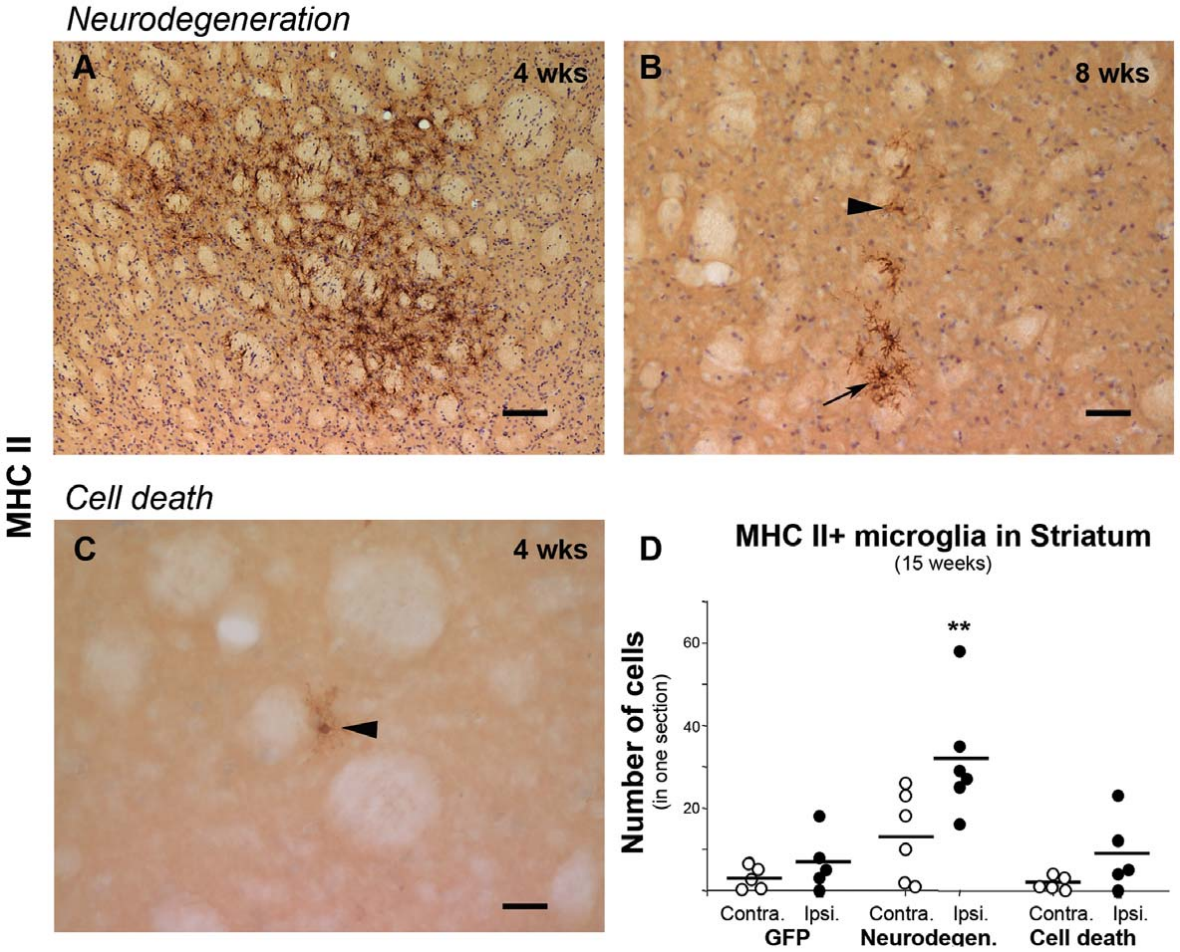
MHC II expression in the Striatum. Photos show representative striatal sections immunostained for MHC II from animals in the α-syn neurodegeneration (**A**–**B**) or cell death group (**C**). In the neurodegeneration group we saw an up-regulation of the molecule throughout the striatum at 4 weeks (**A**). At 8 weeks, expression persisted but had diminished to several isolated cells or small clusters (**B**). In the cell death group, we only observed few isolated weakly stained positive cells at 8 weeks (**C**). Graph shows average (bar) and individual MHC II+ cells numbers found in one striatum section at 15 weeks. These are plotted per animal in each group (Contralateral vs. Ipsilateral) (**D**). One-way ANOVA when significant followed by Tukey-Kramer post-hoc analysis, (**) p<0.01 compared to ipsilateral site of the other two groups.

We also studied the microglia status in the striatum, which is the area that shows the first signs of neurodegeneration and is distant from the injection site. For this we stained for CD3, CD68 and MHC II. Neither apparent up-regulation of CD68 nor presence of CD3+ cells was observed in any group at striatal level (not shown). However, in the α-syn-neurodegeneration group (4 and 8 weeks), numerous MHC II+ microglia clustered throughout the ipsilateral striatum in 50% of the animals (Figure 9A), while in the remaining ones, MHC II expression was of varying degree. Although this did not reach significance at this time point (data not shown), at 15 weeks, the α-syn-neurodegeneration group showed significantly more MHC II+ cells than the other groups (Figure 9D). However at this point, the number of cells and clusters were lower than at earlier points (Figure 9B). In the α-syn-cell death group, only 2 (out of 5) animals at each time point exhibited more MHC II+ cells that in the contralateral side. These were isolated lightly stained MHC II+ cells (Figure 9C). GFP animals only showed occasional lightly stained MHC II+ cells in both ipsi- and contralateral striatum at all time points (not shown).

## Discussion

We have previously shown that overexpression of human α-syn in rat and monkey midbrain results in development of a progressive dopaminergic neuropathology, leading to neuronal loss in SN and fibers in the striatum. This is accompanied by pathological accumulation of α-syn in cell bodies and fibers [41], [42]. In the present study, we believe we were able to mimic both early and late stages of PD: where striatal dopaminergic fiber loss, thickening of the fibers and pathological accumulation of α-syn was observed in both groups; but where α-syn only induced cell loss in the SN in one of the groups. This suggests that α-syn mishandling is happening in both groups and that this results in a neurodegenerative process that begins in the neuronal terminal. We demonstrate that the microglial response is distinct and specific to each time point. Indeed, when α-syn induced cell death microglia acquired a predominant macrophagic phenotype. This correlated with the elevated expression of the phagocytic marker CD68 and recruitment of the adaptive immune system, as judged by the significant presence of CD8+ cells. While α-syn neurodegeneration that did not cause cell death, induced microglia with thin elongated branched dendrites that correlated with high MHC II expression in both SN and striatum. This suggests that, during the initial stages of α-syn induced neurodegeneration, microglia acquire an antigen presenting cell phenotype.

Microglia, as the resident innate immune cells in the CNS, are the surveyors of the brain, constantly moving and scanning their surroundings for damaged neurons, functional state of synapses, plaques and foreign antigens (see review [47], [48]). They do this through cell-cell interactions, pinocytosis of the extracellular matrix and nibbling of other cells membrane [49]. Microglia are extremely sensitive to even small pathological changes, eliciting prompt responses by modulating their number, morphology and cell surface receptors. We observed a marked increase in microglia cell number (Mac1+) in SN when α-syn was overexpressed, which agrees with previous observations in mice during α-syn pathology/neurodegeneration [50], [51], [52], [53], [54]. However, gliosis followed different kinetics depending on the degree of neuropathology: a rapid high magnitude response (neurodegeneration) or a prolonged slower one (cell death). As microglia are a major source of cytokines and chemokines, the extent and magnitude of the response is a key element of regulation that could tip the balance between a beneficial and detrimental response. It is important to note, that when comparing microglial responses one should take into account not only numbers, but also profiles and more importantly the marker used to define microglia. A recent study reported microgliosis in striatum but not in SN in animals over-expressing mutated A53T α-syn via rAAV [55]. Here they used Iba1 as a marker of microglia. Iba1 is a well-documented microglia specific protein that is often used as an alternative marker for Mac1. However, while Iba1 is implicated in Ca binding, Mac1 is an integrin that binds to multiple factors and acts as a subunit of Complement Receptor 3 [56], [57], [58]. This means that we may well be looking at two different scenarios.

A causative relation between extracellular α-syn and activation of microglia *in vitro* has been suggested [26], [29], [59]. In culture, microglia uptakes and processes α-syn with higher efficiency than other brain cells [60]. Furthermore, the efficiency of this process seems to depend on the activation state of the microglia and if α-syn is in monomeric or oligomeric form [60], [61]. This relationship between α-syn and microglia activation has also been reported *in vivo* [50], [51], [62]. So depending on the stage of the disease, the type of α-syn present in the cell may vary, as well as the form it is presented to and/or uptaken by microglia. This could result in α-syn eliciting different microglial responses. In fact, our data suggest that the way α-syn is handled by the cell will determine the type of microglia response. The induction of distinct protein expression patterns and cell differentiation, will ultimately lead to specific cytokine profiles and and cell-to-cell interactions.

In our study the increase in Mac1+ cell number was paralleled by changes in the distribution of the different cell profiles/types of the microglial population. Although at long term the total number of microglial cells return to normal, cell profile/type distribution was different from that observed in either GFP or contralateral control, suggesting a persistent activation of the microglia. In the occurrence of cell death, microglia showed a predominant macrophagic morphology (Type D) that persisted with time, which correlated with elevated CD68+ expression (phagocytic marker). This suggests that above a certain level of α-syn neuropathology the microglial response in the SN becomes detrimental. In fact *in vitro*, co-culture of neurons with macrophagic microglia results in increased α-syn modifications and in turn in dopaminergic cell death [63]. Further supporting this correlation, when α-syn neuropathology was only apparent in the striatum, microglia did not express CD68 and cell death was absent. This differs from a previous work using a similar approach, where in absence of cell death, a significant increase in CD68+ cells was observed at 4 weeks but not at later time points in animals expressing α-syn [64]. Interestingly, in their report and in our hands, CD68 expression peaks at 4 weeks in the GFP group. Thus, CD68 up-regulation at 4 weeks is probably due to the viral vector/injection, a response that is resolved already at 8 weeks in both their and our studies [64]. It is therefore safe to state that the persistence of CD68+ cells is not related to a vector/injection-induced response, but rather to the presence of high cellular levels of human α-syn. It should be noticed as well, that in our approach cell death was complete by 4 weeks since no further decrease of TH+ cells in SN was observed beyond this time point. Thus the persistence of macrophagic microglia, beyond the neurodegenerative process, points to the induction of a long-lasting inflammatory response to human α-syn expression.

In PD patients, it has been observed that microglia express HLA-DR, the human homologue of MHC II [14]. Furthermore, MHC II expression, but not CD68, has been correlated to α-syn deposition in surviving cells in SN from PD patients [38]. MHC II is expressed by antigen presenting cells, which constantly present antigen of extracellular origin to CD4+ T cells. In our *in vivo* model, α-syn induced MHC II expression in the SN independently of the level of neuropathology. However, animals lacking overt pathology in SN showed a more robust MHC II increase, suggesting that its induction is dependent on the presence of neurons expressing α-syn (whose number was higher in the α-syn-neurodegeneration group) rather than cell death. Furthermore, when we investigated the expression of MHC II in the striatum, we observed that only animals with striatal neurodegeneration but not cell death (which exhibited higher density of α-syn+ fibers at all times) showed a consistent up-regulation of the protein. As fibers decreased in number and pathological accumulation of α-syn increased, MHC II expression diminished in these animals. Therefore our data suggest that MHC II expression correlates with the availability of neurons and fibers expressing α-syn.

In PD patients, T cells are found in SN [36], [65] and the ratios between T-lymphocytes are altered [66]. Our data suggest that α-syn can activate the adaptive immune system through MHC II mediation. If endocytosed by microglia, α-syn could be loaded into the MHC II complex and then be presented to CD4+ T cells that will orchestrate the immune response. Indeed, in the MPTP model microglia proliferation has been shown to precede recruitment of T cells, suggesting that involvement of the adaptive immune system is preceded by microglia activation [65]. Accordingly, in our study, α-syn expression in SN led to the recruitment of T cells. The number of T cells observed seemed more relevant in animals presenting cell death, even though MHC II expression was observed in both groups. We could thus anticipate that the type of α-syn presented to the T cell is different in each case, therefore initiating different responses, further supporting the idea that the form of α-syn present in the brain may determine the type of response. Accordingly, nitrated α-syn has been shown *in vivo* to trigger a detrimental immune response that results in neurodegeneration [33]; while vaccination against unmodified α-syn has been shown to be protective [67]. Indeed, although both α-syn expressing groups recruited CD4+ and CD8+ T cells, this recruitment was retarded in the occurrence of cell death, where the CD8 response was markedly higher. There is mounting evidence *in vivo* that different subtypes of T cells, CD4 vs. CD8 [65] and effector T cells vs. regulatory T cells [32], [68], play distinct roles during PD-like progression. Thus one could envisage that depending on the type of microglia presenting antigen, which may be determined by the type of α-syn, different cytokines will be elicited and these will direct the T cell response along different pathways.

Overall, we find that α-syn overexpression in the SN induced a robust early and long lasting microglia activation that was dependent on the presence of α-syn but not on cell death. Indeed, the microglial response observed is an event initiated and sustained by α-syn overexpression that could play a role in the death or survival of the affected dopaminergic neurons. In addition our data suggest the involvement of the adaptive immune system, as shown by MHC II up-regulation and lymphocyte infiltration. While it is tempting to speculate about a possible therapeutic intervention by anti-inflammatory agents targeting the CD68 expressing microglia, at the present time it is unclear what the roles of these two microglia populations are (MHC II+ vs. CD68+) and their relation to the adaptive immune system. Activated microglia has been shown to release pro-inflammatory cytokines such as IL-6 and TNFα that could exacerbate neuronal damage. However, it has also been reported that microglial cells are able to release anti inflammatory cytokines such as IL-10 or neuroprotective molecules such as NT3, NGF and BDNF that could play an important role in neuronal survival (for review see [48], [69], [70]). Changes in cytokines and growth factors in PD patients reveal the existence of a detrimental unbalance between pro-inflammatory deleterious microglia (expressing IL-1, TNFα and IL-6) and protective microglia (releasing NGF, BDNF) [71], [72], [73]. Our data clearly brings to light the existence of two distinct microglial responses dependent on the stage of α-syn induced neuropathology, with MHCII or CD68 up-regulated differentially. Further studies will be required to address the role of the different microglia subpopulations during the neurodegenerative process.

## Supporting Information

Figure S1 Cell association with blood vessels. Representative photos show blood vessels in SN of animals overexpressing α-syn. In animals where cell death was observed, the cells associated with blood vessels (counterstained with cresyl blue) were Mac1 low/neg at 4 weeks (arrowheads in A and B). Mac1+ cells were observed at 4 weeks (C and D) in the α-syn-neurodegeneration group, as well as, at 8 weeks in both α-syn groups (not shown). Note how Mac1+ microglia seem to extend their processes into the vessel (arrows in C) or appear in close association with the vessel lumen (arrowheads in D). Scale: A, B and D, 20 µm (D applies to C).
(3.50 MB TIF)
