# Supplementary figures and images for: Microglia Acquire Distinct Activation Profiles Depending on the Degree of α-Synuclein Neuropathology in a rAAV Based Model of Parkinson's Disease
Source: PLoS One. 2010 Jan 20;5(1):e8784. doi: 10.1371/journal.pone.0008784 (PMC2808388; doi:10.1371/journal.pone.0008784)

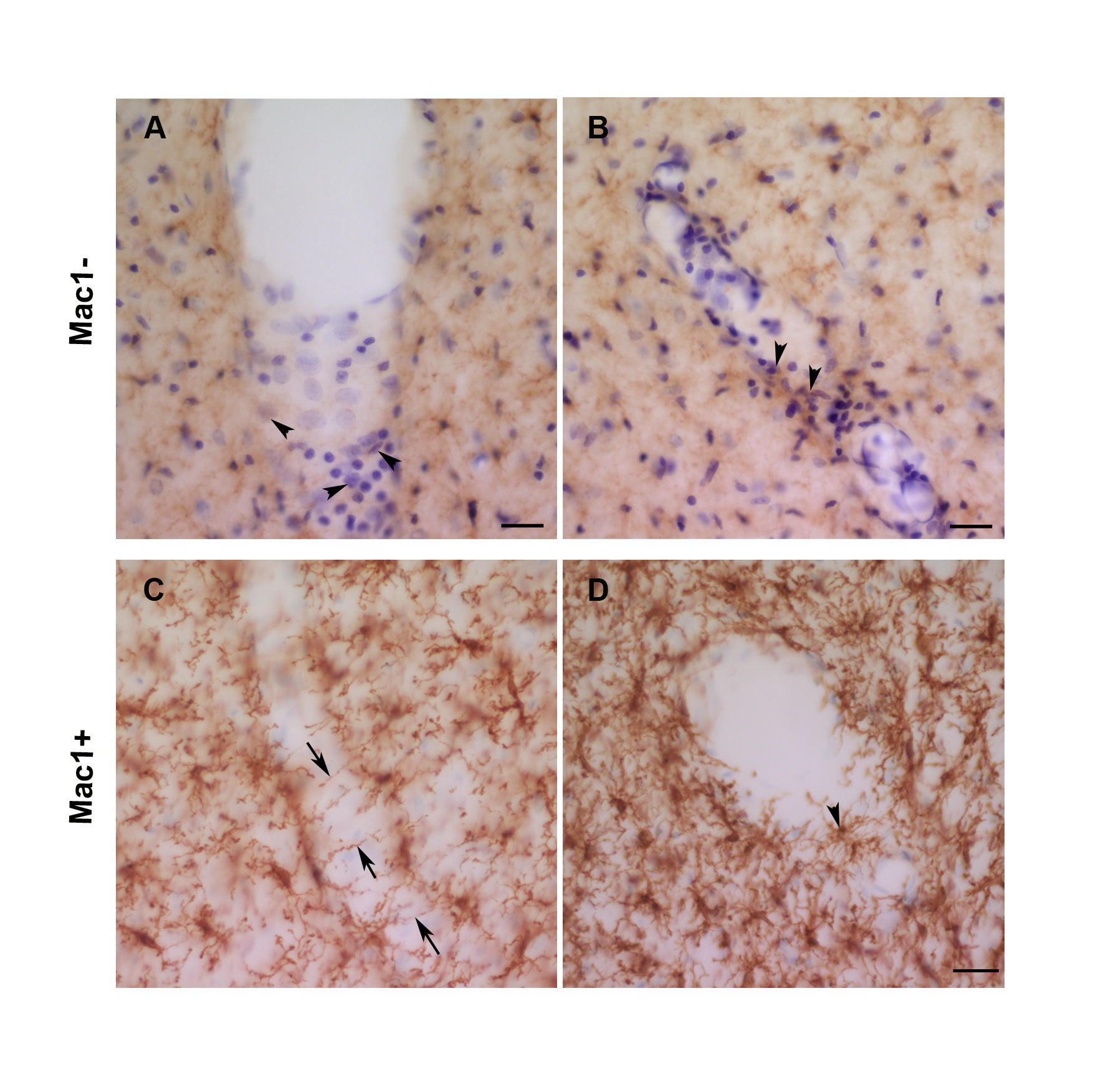

Supplement: Figure S1 — Cell association with blood vessels. Representative photos show blood vessels in SN of animals overexpressing α-syn. In animals where cell death was observed, the cells associated with blood vessels (counterstained with cresyl blue) were Mac1 low/neg at 4 weeks (arrowheads in A and B). Mac1+ cells were observed at 4 weeks (C and D) in the α-syn-neurodegeneration group, as well as, at 8 weeks in both α-syn groups (not shown). Note how Mac1+ microglia seem to extend their processes into the vessel (arrows in C) or appear in close association with the vessel lumen (arrowheads in D). Scale: A, B and D, 20 µm (D applies to C). (3.50 MB TIF) [file pone.0008784.s001.tif]
